# Bradykinin B2 Receptor Signaling Increases Glucose Uptake and Oxidation: Evidence and Open Questions

**DOI:** 10.3389/fphar.2020.01162

**Published:** 2020-08-04

**Authors:** Marcos Fernandes Gregnani, Talita G. Hungaro, Leonardo Martins-Silva, Michael Bader, Ronaldo C. Araujo

**Affiliations:** ^1^ Laboratory of Genetic and Metabolism of Exercise, Departamento de Biofísica, Universidade Federal de São Paulo, São Paulo, Brazil; ^2^ Max-Delbrück Center for Molecular Medicine (MDC), Berlin, Germany; ^3^ Institute for Biology, University of Lübeck, Lübeck, Germany; ^4^ Charité University Medicine, Berlin, Germany; ^5^ German Center for Cardiovascular Research (DZHK), Partner Site Berlin, Berlin, Germany

**Keywords:** bradykinin, kinin B2 receptor, glucose, uptake, oxidation, metabolism

## Abstract

The Kinin B2 receptor (B2R) is classically involved in vasodilation and inflammatory responses. However, through the observation of hypoglycemic effects of Angiotensin-I-Converting Enzyme (ACE) inhibitors, this protein has been related to metabolic glucose modulation in physiological and pathophysiological contexts. Although several studies have evaluated this matter, the different methodologies and models employed, combined with the distinct target organs, results in a challenge to summarize and apply the knowledge in this field. Therefore, this review aims to compile human and animal data in order to provide a big picture about what is already known regarding B2R and glucose metabolism, as well to suggest pending investigation issues aiming at evaluating the role of B2R in relation to glucose metabolism in homeostatic situations and metabolic disturbances. The data indicate that B2R signaling is involved mainly in glucose uptake in skeletal muscle and adipose tissue, acting as a synergic player beside insulin. However, most data indicate that B2R induces increased glucose oxidation, instead of storage, *via* activation of a broad signaling cascade involving Nitric Oxide (NO) and cyclic-GMP dependent protein kinase (PKG). Additionally, we highlight that this modulation is impaired in metabolic disturbances such as diabetes and obesity, and we provide a hypothetic mechanism to explain this blockade in light of literature data provided for this review, as well as other authors.

## Introduction

The Kallikrein-kinin system (KKS) consists of a group of proteins and peptides which play an important role in several physiological functions, such as vasodilation, inflammatory responses, and metabolic adaptations. The precursor of this system is Kininogen, which can be cleaved by Kallikrein to give rise to Kinins, peptides that possess the ability to induce physiological alterations through the kinin B2 receptor (B2R).

These peptides can be further cleaved to generate an agonist for a second receptor, the kinin B1 receptor (B1R). While the B2R is broadly expressed in physiological conditions, the B1R seems to be more important in the inflammatory context (Pesquero et al., 2000; Talbot et al., 2011; Murugesan et al., 2016; Qadri and Bader, 2018). Moreover, kinins can be also cleaved by the Angiotensin-Converting Enzyme (ACE), which generates inactive peptides that can enter the degradation process (Deddish et al., 1996; Dendorfer et al., 1999; Moreau et al., 2005).

The first studies aiming to analyze the relationship between B2R and glucose homeostasis were driven by the evidence of hypoglycemic effects caused by ACE inhibitors (ACEi) on hypertensive patients (Arauz-Pacheco et al., 1990).

These results led the researchers to focus on additional benefits of ACEi for these patients, since glycemic disturbances are commonly found in association with hypertension (Mitchell et al., 1990; Haffner et al., 1998; Juutilainen et al., 2005; Hoffman et al., 2015).

In this context, kinins emerged as the main candidate to be the active agent in this adaptation, since it was observed that ACEi-mediated glycemic effects could not be attributed to decreased Angiotensin II synthesis (Rosenthal et al., 1997).

The articles cited in this review explore the relationship between kinins and glucose homeostasis through two main strategies: analyses of ACEi-mediated effects, and direct effects of kinins on different physiological situations.

Even though the number of publications addressing B2R-modulated glucose metabolism is considerable, the huge variation in the methodologies used becomes a challenge when we intend to combine all aspects into a general picture. Therefore, the purpose of this review is to compile information in this field and synthesize the results to clarify the knowledge in the area, as well as to suggest new directions for future research.

## Methods

We selected articles published between 1990 and 2020 specifically aimed at evaluating the role of B2R in glucose metabolism. For the articles to be included in this review, they should meet the following inclusion criteria: original communications, article data obtained in mammal species, articles in animal models should have some strategy to ensure that treatment effects would be exclusively resulting from B2R signaling, and finally, the articles should be published in English language.

The search was carried out in MEDLINE/PubMed (https://www.ncbi.nlm.nih.gov/pubmed/) and the keywords/terms used were: b2 kinin receptor, b2 knockout mice, bradykinin and Ace inhibitors plus the term “glucose metabolism”. Thus, we had a total of four search combinations: “b2 kinin receptor AND glucose metabolism”; “b2 knockout mice AND glucose metabolism”; “bradykinin AND glucose metabolism” and “Ace inhibitors AND glucose metabolism”.

**Figure 1:**
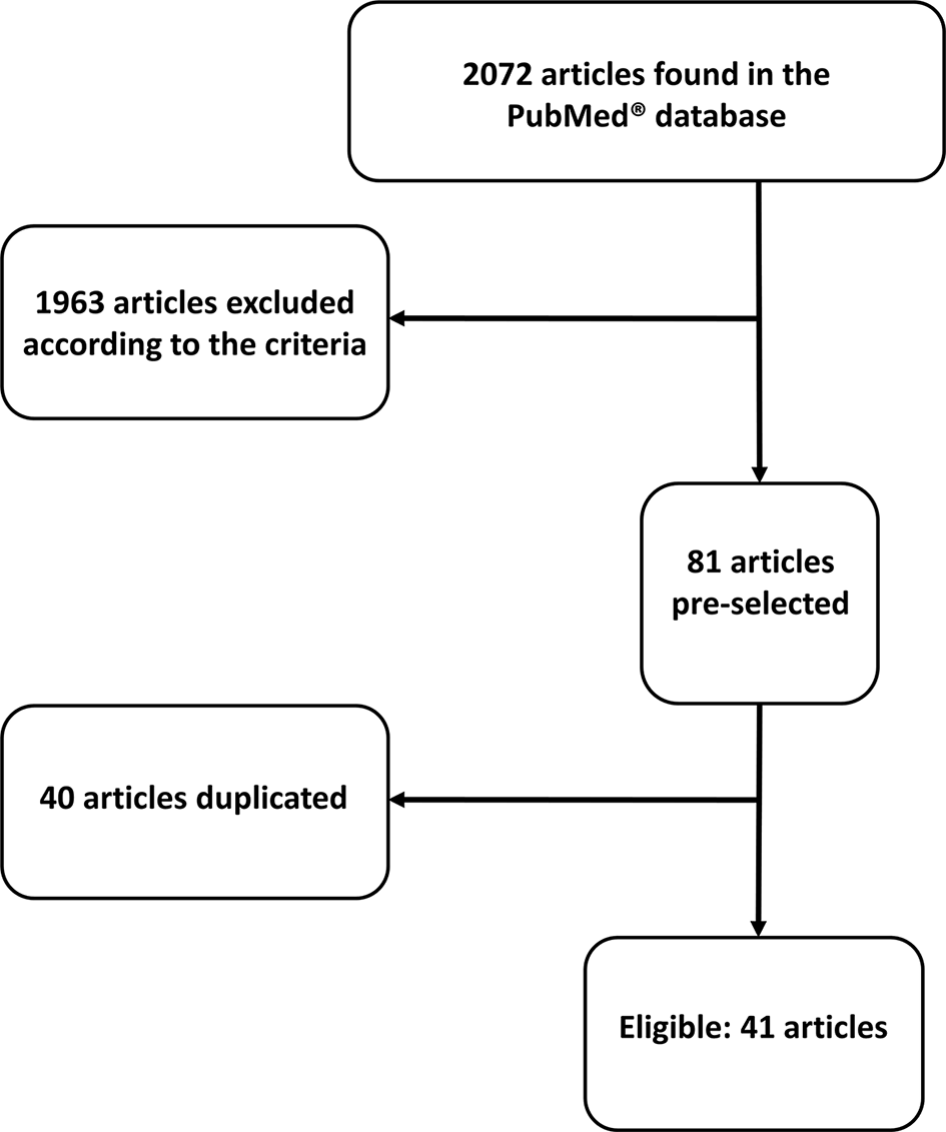
Flow chart showing search process.

Upon abstract analysis by two independent investigators, duplicated articles were excluded, as well as those which did not fulfill our criteria. Ultimately, 41 articles were selected. More details about the search process can be found in **Figure 1** and **Supplementary Materials**. We also provide the summarized findings of included articles in **Table 1**.

**Table 1:** Summarized data of selected articles.

Article	Sample	Tissue	Methods	Findings
Uehara et al., 1994	Dogs and humans	Sistemic analisys	Chronic (*in vitro*) ACE inhibitor and HOE 140 treatments	ACEi improved insulin sensitivity and this result seemed to be related to BK.
Tomiyama et al., 1994	Rats - SHR	Sistemic analisys	Chronic (*in vivo*) Enalapril, losartan and HOE 140 treatments	The improvement in insulin sensibility by ACEi was kinin dependent (B2R).
Yang and Hsu, 1995	Rats	Pancreas (perfused)	Animals treated with BK (1uM) and HOE 140 (0.1uM) - acute treatment	BK increased insulin release and may play a physiologic role in this regulation.
Henriksen and Jacob, 1995	Rats - Zucker	Skeletal muscle	Captopril and HOE 140 - acute and chronic treatments *in vivo*	Captopril ameliorated glucose uptake in skeletal muscle through BK.
Henriksen et al., 1996	Rats - Zucker	Epitrochlearis muscle	ACEi (acute and chronic *in vivo* administration) and HOE 140.	ACEi increased insulin sensitivity. The acute effect was abolished by pretreatment with HOE 140.
Chen et al., 1996	Rats - Wistar	Sistemic analisys	HOE 140, alacepril and TCV-116 (chronic *in vivo* treatments)	HOE 140 did not improve glucose tolerance suggesting that the effects of ACE inhibitors were not due to kinins.
Zuccollo et al., 1996	Mice - Male C57BL/Ks mdb	Sistemic analisys	STZ injection (40mg/kg) for 5 days and consecutive treatment with B1R or B2R antagonist.	HOE 140 did not show effects. B1R antagonist normalized glycemia and diuresis, protein, nitrite and kallikrein excretion.
Saito et al., 1996	Hamster	Cells	BK and HOE 140 acute *in vivo* treatments	BK increased calcium and induced the insulin release. HOE 140 prevented the effect.
Nuutila et al., 1996	Humans	Sistemic analisys	BK and insulin acute *in vivo* treatments	BK increases skeletal muscle blood flow but not muscle glucose uptake *in vivo*.
Shimamoto et al., 1996	Rats	Sistemic analisys	Delapril (ACEi) and HOE 140 - chronic *in vivo* treatments	The hypoglycemic effects of ACEi were not related to BK in this model.
González et al., 1997	Rats (streptozotocin-diabetics rats)	Liver and uterus	Chronic *in vivo* administration of HOE-140 and L-NAME	Suggests that N.O production induced by B2 kinin receptor would be responsible for some of STZ damage in these organs.
Yang et al., 1997	Rats	Pancreas	BK - acute *in vivo* treatment	BK increased insulin concentration and had a similar transitory effect on glucagon.
Kishi et al., 1998	Rat, Mouse and Hamster cell lines	L6: rat myotube; 3T3-L1 mouse embryo adipocytes, CHO (hamster ovary) cells expressing c-myc.	BK - acute treatment	BK increased GLUT 4 translocation and the rate of glucose uptake in a dose dependent way.
Laine et al., 1998	Humans (control and obese group)	Sistemic analisys	BK and insulin - acute *in vivo* treatments	Glucose uptake *via* insulin was lower in obese individuals. BK did not show effect on this parameter.
Caldiz and de Cingolani, 1999	Rats (normal and hypertense)	Cells - Adipocytes	Enalapril, insulin and HOE 140 - chronic *in vivo* treatments	HOE 140 inhibited the glucose uptake induced by enalapril.
Damas et al., 1999	Rats	Sistemic analisys	Captopril and HOE 140 - *in vivo* acute treatments	Animals knockout to kininogen showed impaired functions in relation to glucose metabolism as well as normal rats treated with HOE 140.
Henriksen et al., 1999	Rats-Zucker	Epitrochlearis muscle	Acei, Bk, HOE 140 and L-NAME,insulin	Acei and/or Bk increased insulin-induced glucose uptake, HOE104 and L-NAME abolished the effects.
Zuccollo et al., 1999	Mice - Male C57BL/Ks mdb	Sistemic analisys	STZ injection (40mg/kg) for 5 days with or without HOE140 or ACEi	HOE140 and ACEi did not have effect in glycemia.
Kudoh and Matsuki, 2000	Rats	Cell - L6 (skeletal muscle/myoblast)	Bk, HOE 140 and enalapril - acute *in vitro* treatments	ACEi induced glucose uptake *via* B2 kinin receptor pathway.
Taguchi et al., 2000	Humans and rats	Skeletal muscle	Exercise and HOE 140 - acute *in vivo* treatments	Glucose influx and GLUT4 translocation were up regulated after exercise. These effects were abolished after HOE 140 treatment.
Frossard et al., 2000	Humans	Skeletal muscle	Enalaprilat, Losartan and BK - acute *in vivo* treatments	The effect in arterial interstitial gradient for glucose was related to BK.
Damas et al., 2001	Rats	Sistemic analisys	BK, HOE 140 and L-NAME - acute *in vivo* treatments	BK increased glucose; HOE 140 and L-NAME abolished this effect. In specific conditions, BK also increased insulin levels.
Shiuchi et al., 2001	Mice - FVB/N	Skeletal muscle and adipose tissue	BK, HOE 140, Leptin and L-NAME - acute *in vivo* treatments	The glucose uptake induced by leptin was inhibited in skeletal muscle by HOE 140 and L-NAME
Duka et al., 2001	Mice (WT and B2KO)	Sistemic analisys	Captopril chronic treatment - *in vivo*	B2KO animals showed higher basal insulin as well as lower sensibility to this hormone.
Arbin et al., 2001	Rats - Zucker	Sistemic analisys	ACE inhibitor, NEP, HOE 140 and L-NAME treatments	ACEi improved insulin mediated glucose disposal (IMGD). ACE/NEPi improved IMGD more effectively. HOE 140 and L-NAME inhibited the insulin sensitivity index induced by ACE/NEPi
Dumke et al., 2002	Rats	Skeletal muscle	HOE 140 and insulin - acute *in vitro* treatments	HOE 140 did not change the glucose uptake induced by insulin.
Schiuchi et al., 2002	Mice (WT and KK-Ay/Ta diabetic mice) and cells (L6 skeletal muscle lineage)	Systemic analisys and Cells	Acei, L-NAME, HOE 140-14 days *in vivo* treatment. Insulin acute treatment	Acei improved insulin resistance, which was blocked by HOE140 and L-NAME treatment. The effects were due increased skeletal muscle glucose uptake *via* GLUT4 translocation after insulin treatment
Cahová et al., 2003	Rats (HHTg and control Wistar rats)	Sistemic analisys	Captopril (chronic) and HOE 140 (acute) - *in vivo* treatments	Captopril and HOE 140 showed effects only in control rats.
Wang et al., 2003	Rats - Zucker	Sistemic analisys	Omaprilat, ramipril, losartan and HOE 140 - *in vivo* chronic treatments	Higher glucose uptake and lower glucose production dependent of BK.
Schaffer et al., 2004	Rats-Zucker	Sistemic analisys	Vasopeptidase inhibitor,icatibant (B2R inhibitor)	There were no effects in glycemic status
Montanari et al., 2005	Rats	Sistemic analisys	STZ, Kallikrein gene delivery and HOE 140	Kallikrein gene delivery improved fat pad and skeletal muscle weight, effects abolished by HOE 140
Beard et al., 2006	Rats	Cells - Adipocytes	BK and HOE 140 - acute *in vitro* treatments	BK enhanced insulin sensitivity in adipocytes *via* NO pathway. Insulin increased glucose uptake through IRS-1 phosphorilation.
Tan et al., 2007	Mice (WT and B2KO)	Sistemic	STZ (50 mg/kg) 3-5 days	Genotype did not cause any difference in glycemic status
Pretorius and Brown, 2010	Humans - control, overweight and obese	Sistemic analisys	BK e L-NAME - *in vivo* acute treatments	BK increased glucose uptake and the L-NAME treatment reversed this phenotype
Schweitzer et al., 2011	Mice - WT and B2KO C57BL/6	Skeletal muscle	Acute exercise	B2R was not essential for glucose uptake after exercise
Barros et al., 2012	Mice - Ob/Ob, ObB2KO and WT C57BL/6	Sistemic and hepatic analisys	BK - acute *in vitro* treatment	The absence of B2R and the obesity model worsened the animal phenotype. BK had a protective effect in liver.
Iozzo et al., 2012	Humans - control and obese group	Adipose tissue	BK and insulin - acute *in vivo* treatments	BK increased glucose uptake *via* insulin in control group
Simões et al., 2013	Humans - control and type 2 diabetes	Sistemic analisys	Exercise - 3 sessions of different kinds of moderate intensity aerobic exercise	BK was up-regulated in control group and Des-Arg9-BK up-regulated in diabetic group.
Reis et al., 2015	Mice - WT and B2RKO C57BL/6	Sistemic and skeletal muscle analisys	High fat diet and exercise - chronic *in vivo* treatments	B2RKO mice had higher muscle glucose uptake,as well as increased glygogen synthesis in insulin stimulated condition
Asano et al., 2017	Humans - control and type 2 diabetes	Sistemic analisys	Acute aerobic exercise on a cycle ergometer in different intensities	BK was increased 45min after exercise. However, no relation with the decrease of glucose and insulin was found
Frigolet et al., 2017	Rats	Cells - Adipocytes	BK and insulin - acute *in vitro* treatments	BK increased insulin effects by boosting signaling pathway and inhibiting negative feedback

Due to a large variation in the aim of the studies, we divided the results into sections for best understanding.

### Systemic Researches

Regarding systemic adaptations, the effects of B2R signaling on glycemic status seem to be related to sympathetic drive in anesthetized rats. The constant infusion of bradykinin (BK) caused an increase in glucose blood content, which was abolished when beta-blockers were administered concomitantly. On the other hand, in adrenalectomized rats, B2R induced increased insulin secretion, as well as reduced blood glucose levels, showing that the sympathetic drive can change the B2 kinin receptor effects (Damas et al., 2001).

When kininogen (kinins precursor)-deficient Brown-Norway Katholiek rats were employed to study the role of kinins in glucose homeostasis, it was shown that these animals had glucose intolerance, and delayed insulin response compared with the control rats. When the authors made a screening to elucidate the reasons for these results, they found that the absence of B2R signaling was the source of the disturbances observed (Damas et al., 1999).

Also, SHR rats (genetically hypertensive rats) were sensitive to B2R signaling concerning glucose uptake. When these animals were treated with Enalapril for 3 weeks, the glucose uptake was improved, in addition to benefits related to arterial pressure.

Interestingly, only the adaptations in glucose metabolism were B2R-dependent (Tomiyama et al., 1994). Another genetic model in which the B2R signaling showed positive effects was an obesity model in rats (obese Zucker rats).

In this situation, ACEi-mediated acutely increased B2R signaling improved the glucose sensitivity measured by hyperinsulinemic-euglycemic clamp.The positive effects induced by B2R activation were mediated by nitric oxide (NO) production (Arbin et al., 2001; Wang et al., 2003).

In the same model, although glucose uptake was not improved by ACEi plus B2R signaling, treatment with kinin B2 receptor antagonist caused weight gain in these animals, suggesting an important effect mediated by this pathway in keeping a stable energetic balance (Schaffer et al., 2004).

Dietetic interventions were also used to evaluate the role of B2R in glucose metabolism.

After a 4-week fructose intervention in rats, 2 weeks of treatment with the ACEi Delapril reversed hyperglycemia and insulin resistance, but this was independent of B2R signaling (Shimamoto et al., 1996).

Corroborating these results, in an 8-week fructose intervention, similar results were found, since ACEi treatment decreased the body weight of the rats, without interference of B2R signaling. However, when the authors blocked this receptor, they observed a worsened insulin resistance in the fructose-treated animals, suggesting independent and likely synergistic actions of ACEi and B2R signaling (Chen et al., 1996).

In a type 1 diabetes model (induced by STZ injection) mice treated with B2R agonists or antagonists for 13 days showed no effect related to glycemic homeostasis mediated by this signaling pathway (Zuccollo et al., 1996; Zuccollo et al., 1999; Tan et al., 2007).

In rats, the inhibition of B2R signaling caused improvements in metabolic outcomes: glycaemia and triglycerides decreased in animals treated with B2R inhibitors, as well as NO synthesis inhibitors (González et al., 1997), suggesting that, in this context, B2R/NO signaling negatively modulates the metabolic adaptations, maybe due to inflammatory environment.

On the other hand, in type I diabetic rats, an intervention consisting of human Kallikrein I gene delivery was able to improve blood glucose, decrease epididymal fat pad weight and increase gastrocnemius weight. Although B2R did not influence glycemic improvements, this signaling was responsible for the reductions in epididymal fat content and skeletal muscle mass, contributing with the better metabolic profile (Montanari et al., 2005).

Using ACE inhibitors initially in dogs, Uehara et al. (1994) showed that the increased insulin sensitivity caused by Captopril was dependent on B2R signaling, and this result was repeated in diabetic animals, suggesting that maybe some intra-species differences can be observed in relation to outcomes in diabetic state. Also, the authors were able to repeat these observations in non-diabetic and diabetic human subjects, since ACEi injection improved insulin sensitivity in both patient groups, and this adaptation was observed along with an increase in plasma BK content.

### Skeletal Muscle

The skeletal muscle has been shown as a tissue with a huge role in several physiologic responses, some of them related with glucose metabolism regulation. However, in this section our focus will be the glucose uptake. It is already well known that there are “brain-periphery” circuits which control glucose homeostasis through feedback mechanisms between the brain and liver, adipose tissue, pancreas, and skeletal muscle (Balthasar et al., 2004; Dhillon et al., 2006; Leinninger et al., 2009; Kuliczkowska-Plaksej et al., 2012).

It seems that B2R signaling may take part in this process, since it has been shown in mice that B2R signaling in skeletal muscle was related to the central action of leptin, being part of responses aimed to promote glucose uptake. Leptin induced bradykinin release in the bloodstream, which activates B2R signaling in epitrochlearis muscle. This activation increased NO production and induced skeletal muscle glucose uptake in an insulin-independent pathway, suggesting additive effect of B2R and insulin signaling (Schiuchi et al., 2001).

Also, in healthy rats, B2R signaling was effective in modulating glucose uptake in epitrochlearis muscle, without affecting muscle contraction (Dumke et al., 2002). In a rat genetic model of obesity, a 14-day treatment with ACEi was able to improve glucose uptake in epitrochlearis, and the effects induced by B2R/NO signaling were observed only in the presence of insulin, suggesting a cooperative, but not independent, role for this pathway (Henriksen and Jacob, 1995; Henriksen et al., 1996; Henriksen et al., 1999).

In a genetic model of type 2 diabetes, an improvement in skeletal muscle glucose uptake was also observed after treatment with ACEi for 8 weeks in mice. These effects in hindlimb muscles were, again, mediated by B2R/NO independent of insulin and, as a mechanism for increased glucose uptake, the authors showed increased GLUT4 translocation towards cell membrane (Schiuchi et al., 2002).

Kinin B2 receptor knockout animals (B2KO) provided some insights on the role of these receptors in glucose homeostasis. The B2KO mice had increased basal glycaemia and decreased basal insulin content, suggesting that the absence of B2R causes disturbances in insulin secretion. Furthermore, these animals had impaired insulin-induced glucose uptake in soleus, with no changes in insulin-mediated Akt phosphorylation (Schweitzer et al., 2011).

Since it has also been shown that an insulin injection was able to increase B2R expression in skeletal muscle of wild-type mice (Duka et al., 2001), there is the hypothesis that kinin B2 receptor exerts an adjuvant role in insulin-induced glucose uptake.

When we look to dietetic approaches, the results are not so homogeneous. While in rats submitted to a diet with an excessive sucrose content ACEi could prevent insulin resistance through B2R signaling and improvement of glycogen storage in soleus (Cahová et al., 2003), in an HFD model, B2KO mice were refractory to weight gain when compared with wild-type mice, and had better insulin sensitivity and increased glycogen synthesis in the soleus. However, it is interesting to mention that these mice had glucose intolerance (again likely due to decreased insulin secretion) (Reis et al., 2015).

In healthy humans, acute treatment with ACEi was able to increase vastus medialis glucose uptake during a hyperinsulinemic/euglycemic clamp, mediated by B2R activation, corroborating the data from animal models (Frossard et al., 2000).

Similar to what happened in animals, these data in healthy humans predicted a positive effect of this pathway in humans with metabolic diseases. In obese humans, a forearm BK injection resulted in increased forearm glucose uptake, mediated by increased NO production, corroborating the participation of the B2R/NO pathway in skeletal muscle glucose uptake (Pretorius and Brown, 2010). However, these data contrasted with previous studies in healthy and obese humans, where the concomitant administration of BK and insulin had opposite effects. Although BK increased vasodilation in leg muscles, the glucose extraction was decreased in both obese subjects and healthy controls (Nuutila et al., 1996; Laine et al., 1998).

Besides entire organism models, *in vitro* assays on isolated tissues are also useful to explore the mechanisms behind the phenotypic alterations observed in a controlled environment. Regarding B2R signaling in L6, a skeletal muscle lineage, ACEi treatment increased glucose uptake *via* GLUT4 translocation independent of insulin (Schiuchi et al., 2002).

On the other hand, (Kudoh and Matsuki, 2000) observed B2R effects only in presence of insulin and these were dependent on calcium increases and activation of phospholipase C, instead of the canonical insulin pathway (Kudoh and Matsuki, 2000). These data open new avenues for research on the interaction between insulin and G-coupled receptors in this tissue.

### Adipose Tissue

In addition to skeletal muscle, adipose tissue is also an important focus of kinin receptor and glucose homeostasis studies, since this tissue also has a remarkable capacity for glucose storage and participates in the body glucose control.

SHR rats have impaired glucose uptake and decreased lipogenesis rate. The treatment with ACEi increased glucose uptake mediated by insulin in adipose tissue and consequently improved the glucose sensitivity. Interestingly, the treatment did not change the lipogenesis induced by insulin (Caldiz and de Cingolani, 1999) suggesting that B2R signaling can induce glucose oxidation in adipose tissue.

In rats which developed adipose tissue insulin resistance after being submitted to a sucrose-rich diet, treatment with ACEi during the diet protocol was able to protect rats from impairments on glucose uptake. Lipid storage rate was decreased by B2R signaling only in the presence of insulin (Cahová et al., 2003).

Further information on the glucose uptake mechanisms triggered by B2R pathway in adipose tissue can be obtained from *in vitro* approaches. The key adaptation mediated by B2R signaling was the translocation of GLUT4 to the membrane, which was completely independent of insulin in the NIH3T3cell line.

Furthermore, in these cells, B2R signaling was able to decrease lipogenesis in the presence of insulin, but increased it when administered alone (Kishi et al., 1998). In healthy rat adipocytes, however, B2R signaling improved glucose uptake only in the presence of insulin.

The B2R/NO pathway mediated these responses by triggering GLUT4 translocation to the membrane through the inhibition of the negative feedback by insulin triggered by the JNK pathway (Beard et al., 2006). The mechanism involves PKG activation by B2R signaling, which inhibits JNK/ERK pathways through modulation of MKP-5, a Map-kinase dephosphatase protein (Frigolet et al., 2017).

In humans, the ability of B2R signaling to increase adipose tissue glucose uptake was observed only in healthy subjects submitted to a BK injection, but not in obese subjects. However, the vasodilation was seen in both groups (Iozzo et al., 2012).

### Pancreas

The pancreas is an important source of kallikrein and, in addition, it is a central organ in glucose homeostasis through insulin and glucagon secretion. Accordingly, Yang and Hsu (1995) decided to investigate alterations in B2R-modulated insulin secretion. These authors observed an increase in insulin secretion mediated by B2R activation alone, as well as an amplification of glucose stimulated secretion. Since the authors used the whole pancreas in the experiments, it was not possible to distinguish between vascular effects and direct effects on pancreatic beta cells.

*In vitro* studies suggested that the acute effects on the insulin response by B2R pathway are due to calcium modulation. Bradykinin stimulated insulin secretion in basal conditions in a hamster beta cell line *via* increased reticular calcium release (Saito et al., 1996). Furthermore, in the RINm5F line, B2R signaling modulated cellular calcium efflux *via* opening of channels in addition to modulating the endoplasmic reticulum flux. The effects were mediated by phospholipase C and consequent IP3 intracellular increase (Yang et al., 1997).

### Liver

The effects of B2R signaling on liver glucose homeostasis modulation were evaluated only in disease models. In leptin- deficient mice (ob/ob mice), the additional knockout of B2R (generating ob/ob/B2KO mice) led to impaired glycaemia compared with ob/ob mice. While a decrease in glycaemia could be seen in control animals, ob/ob/B2KO mice were not able to reproduce this phenotype due to the increased gene expression of key hepatic gluconeogenesis enzymes, which leads to a sustained hyperglycemia (Barros et al., 2012).

In rats with type 1diabetes, the B2R/NO pathway seems to have an important role, but promoting negative effects. The inhibition of this pathway improved metabolic parameters, such as triglycerides content in the liver and insulin sensitivity. Although hepatic glucose oxidation was not affected, these data suggest that in this model B2R signaling has an inflammatory role (González et al., 1997).

### Exercise

Physical exercise is an interesting model to evaluate stress responses in organism in several contexts. Also, physical exercise has been presented as an important adjuvant in the therapy of diseases such as obesity and diabetes (Gillen et al., 2012; Bohn et al., 2015; Pedersen and Saltin, 2015; Freitas et al., 2018; Gorostegi-Anduaga et al., 2018). Starting from the assumption that B2R is able to modulate glucose uptake under resting conditions, some authors have also evaluated the action of this receptor during physical exercise, which is a classical way to stimulate glucose oxidation. This could be of great importance in normal subjects, but even more in those that have some glucose disturbances such as diabetes and obesity.

After a 60 min treadmill session of moderate intensity, B2KO mice showed no differences in relation to controls. In both groups, similar patterns of glucose uptake and insulin secretion were observed (Schweitzer et al., 2011).

These results contrast with those presented by Reis et al. (2015), where B2KO mice showed improved exercise performance, as measured in both a progressive treadmill exercise and a swimming test.

The better adaptations of these animals in relation to controls were related to a “shift” in skeletal muscle fibers from Type IIa to Type I. These results suggest that B2R receptor can be an inducer of glycolitic metabolism, which is not beneficial during endurance physical exercise practice.

On the other hand, in rats with different “glycemic status”, B2R seemed to have a role in glycemic responses after a 60 min swimming protocol. Healthy animals, as well as diabetic animals with controlled glycaemia, had an increase in serum BK content along with decreased serum glycaemia.

The uncontrolled glycaemia group had a different phenotype: no increase in BK concentration after swimming protocol and increased glycaemia (Taguchi et al., 2000). These results suggest that the previous glycemic status is important to BK release, and B2R signaling. The mechanism underlying these results was identified as increased GLUT4 translocation in hindlimb skeletal muscles of these rats, mediated by increased phosphorylation of PI3K in response to insulin injection.

These results were corroborated in humans by the observation of increased BK in both healthy control and controlled type 2 diabetes patients, after a 20 min cycloergometer session, in opposite to patients with poor glycemic control, who could not react with the same adaptation (Taguchi et al., 2000).

Also, in a moderate intensity aerobic training, it was found that diabetic individuals have impaired kallikrein, BK, and NO release compared with healthy subjects, supporting a relationship between glycemic disturbances and poor availability of these key compounds for the promotion of benefits related to insulin sensitivity (Simões et al., 2013).

The intensity of training modulated glucose uptake response in different ways. In both diabetic patient groups, increased BK was observed only after a session at 80% of maximum capacity, while at 120% there was no increase in the BK content. Since glycaemia was decreased after both sessions, glycaemia was not correlated with bradykinin responses (Asano et al., 2017).

## Discussion

**Figure 2:**
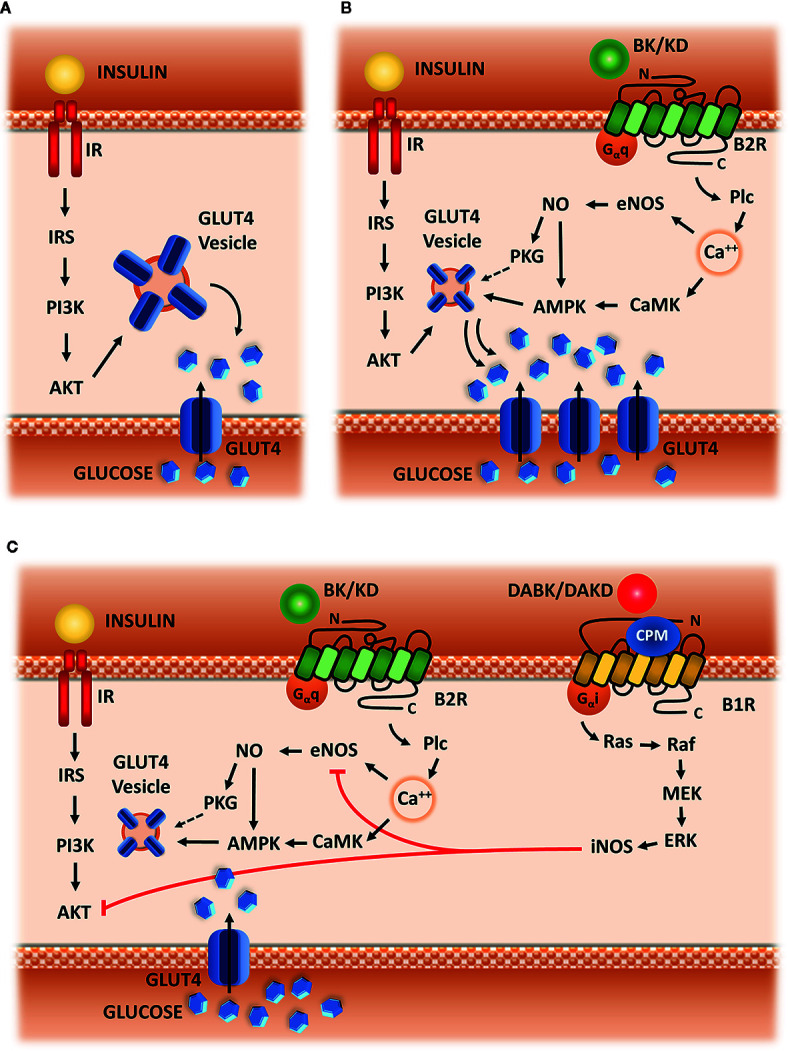
Hypothetic scheme. **(A)** Insulin signaling. Insulin-mediated glucose uptake in adipose tissue and skeletal muscle, which is triggered by IRS-1 phosphorylation, followed by activation of PI3k and AKT, leading to GLUT-4 translocation to membrane. **(B)** Kinin signaling *via* B2R. Signaling starts with Plc activation, followed by calcium release from intracellular storage, CAMK activation, eNOS phosphorylation and NO synthesis, leading to AMPK and PKG activation, and subsequently to increased GLUT4 translocation. **(C)** Kallikrein-Kinin System in glucose uptake. B1R is up-regulated in metabolic disturbances, as well as CPM enzyme, converting B2R agonists (BK or KD) into B1R agonists (DABK or DAKD). B1R pathway starts with the activation of G-alpha-i and beta-gamma subunit, leading to the activation of the following kinases: Src, Ras, Raf, MEK, and ERK.ERK activate iNOS leading to exacerbated NO production. Increased NO impairs glucose uptake, blocking eNOS and AKT activity.

When we analyzed studies involving B2R signaling and metabolism, we identified a pattern of response related to glucose uptake in both adipose tissue and skeletal muscle. In both cases, it is well established that B2R signaling improves glucose entering into cells. This effect seems to be an amplification pathway for insulin-induced glucose uptake (Henriksen and Jacob, 1995; Kishi et al., 1998; Caldiz and de Cingolani, 1999; Henriksen et al., 1999; Schiuchi et al., 2001; Schiuchi et al., 2002; Cahová et al., 2003; Beard et al., 2006; Frigolet et al., 2017), which is a pathway that starts with IRS-1 phosphorylation after insulin binding to its receptor IR. Thereafter, there is phosphorylation of PI3K and Akt, leading to GLUT4 translocation (Cheatam et al., 1994; Sano et al., 2003). This mechanism can be seen in **Figure 2A**.

However, there is still no answer for an important question about the synergic effect between insulin and B2R: after glucose uptake, is the glucose delivered to cell storage in the presence of B2R activation similarly to what happens when only insulin is on charge? The results of our review, along with some previous knowledge about signaling pathways induced by the B2R allow us to raise a hypothesis.

The vast majority of articles selected for this review show that B2R signaling is linked to eNOS activation and NO production. These data led us to conclude that B2R modulation of glucose uptake is dependent on calcium influx, which activates eNOS *via* Plc, PKC and CaMK dependent phosphorylation (Cruzblanca et al., 1998; Kudoh and Matsuki, 2000; Kim et al., 2006; Lee et al., 2015; Kim et al., 2016). Therefore, NO through S-nitrosylation of AMPK, increases glucose uptake by stimulating GLUT4 translocation to the membrane (Fryer et al., 2000; Lira et al., 2007) (**Figure 2B**).

Through AMPK activation, NO would also be able to direct glucose towards a different direction compared with the classical actions of insulin. Indeed, at physiological concentrations, NO is described as a factor that increases glycolysis and decreases insulin-induced glycogen storage in skeletal muscle, in addition to being able to decrease insulin-mediated lipogenesis (Young et al., 1997).

Additionally, there are reports in the literature describing that the overexpression of eNOS is able to protect mice from diet-induced obesity, mainly through mechanisms linked to increased metabolism in adipocytes, blunting the hypertrophy of these cells (Sansbury et al., 2012).

Furthermore, NO (from eNOS) acts through increases in cGMP content, resulting in the activation of PKG, which can directly activate oxidation pathways in adipose tissue through positive modulation of Hormone Sensitive Lipase and Perilipin (Young and Leighton, 1998).

Accordingly, Frigolet et al. (2017) showed an improved glucose uptake mediated by the B2R/NO pathway through the activation of PKG in adipose tissue.

Indeed, several results in this review corroborate a shift of glucose fate induced by B2R signaling, after synergic action with insulin in order to induce glucose uptake. This signaling pathway is able to improve body weight or maintain it stable (Schaffer et al., 2004; Montanari et al., 2005), and decrease the lipid storage induced by insulin (Kishi et al., 1998; Caldiz and de Cingolani, 1999; Cahová et al., 2003), strongly suggesting that B2R/NO pathway is an important modulator of glucose oxidation in insulin sensitive tissues. Based on these previous studies, we suggest that B2R/NO signaling can increase substrate oxidation along with insulin, maybe also in an independent way. These mechanisms are summarized in (**Figure 2B**).

Concerning B2R signaling and glucose metabolism, it is also worth mentioning that a disturbed metabolic environment (obesity/insulin resistance and/or diabetes) leads to a blockage of the B2R/NO pathway with respect to glucose uptake (Zuccollo et al., 1996; González et al., 1997; Zuccollo et al., 1999; Tan et al., 2007).

It is interesting to mention that these alterations are observed in human patients with type 2 diabetes, similarly to what happens in animal models of type 1 diabetes, which strengthens our observations that glycemic disturbances are able to blunt B2R signaling in basal situations, as well as endogenous BK response to physical exercise (Taguchi et al., 2000; Simões et al., 2013).

We hypothesized that low grade inflammatory processes, which are common in these pathologies may be involved in these effects. They are mediated by an increased fat content in adipose tissue and liver, which leads to the activation of immune cells. In addition to inflammation at local level, these tissues become a source of cytokines, which are secreted and spread the inflammatory stimulus to other tissues (Cox et al., 2015; Lopez et al., 2016; Borges et al., 2018).

Among the players that induce and amplify these processes, we can also find NO, which we have mentioned earlier in this text as a protector factor in physiological situations (Yang et al., 2015; Ghasemi and Jeddi, 2017; Liu et al., 2019).

However, if we compare the mechanism of synthesis of this compound in pathological and physiological situations, we can see an important difference. While in homeostatic situations the main pathway of NO production is through eNOS, in pathological situations it is produced by iNOS. This enzyme has a mechanism of activation which is independent of calcium signaling, as well as an increased capacity NO synthesis and long lasting activation (Stuehr et al., 1991; Yui et al., 1991). This results in exacerbated NO production, which can cause systemic insulin resistance as a result of impaired glucose uptake in skeletal muscle and adipose tissue. The mechanism involves S-nitrosylation of IRS-beta and AKT, which interferes with glucose uptake, leading to a decrease in muscle glycogen content (Carvalho-Filho et al., 2005; Carvalho-Filho et al., 2006; Pauli et al., 2008; Carvalho-Filho et al., 2009) and inhibits anti-lipolytic action of insulin in adipose tissue (Ovadia et al., 2011). Besides that, the excessive NO production *via* iNOS inhibits eNOS activity, thereby blocking the main B2R effector (Santhanam et al., 2007; Kim et al., 2009).

Furthermore, our suggested mechanism involves a second duality in addition to NO different role depending on the context and its availability in intracellular compartment. We hypothesized that the source of these disturbances is also part of KKS: B1R activation. As previously mentioned, this receptor has a small expression in homeostatic situations and is upregulated in pathologic situations, including metabolic diseases. This increased expression has functional implications, such as loss of insulin sensitivity and increased production of reactive oxygen species (Dias et al., 2010). The signaling pathway induced by B1R activation implies activation of G-alpha-i protein (Brovkovych et al., 2011), leading to increased activity of iNOS enzyme (Dias and Couture, 2012) in addition to increased NADPH-oxidase activity (Dias et al., 2010; Haddad and Couture, 2016). Besides that, the role of type I Kininases (carboxypeptidases M and N) in directly improving the function of B1R signaling pathway, as well as amplifying those effects through B1R expression up-regulation, is well described in the literature (Zhang et al., 2011; Zhang et al., 2013). Indeed, CPM cellular expression is also up-regulated in insulin resistance and the inhibition of its activity through the experimental drug *Mergetpa* has positive effects similar to those observed when B1R was blocked (Haddad and Couture, 2017). Additionally, another experimental protocol with the same drug corroborates our hypothesis, since the authors showed increased vascular reactivity mediated by BK after the inhibition of type I Kininases (Salgado et al., 1986). Based on these evidences, we hypothesized that the negative or inconclusive results after BK administration in human/animal interventions with obese or diabetic subjects can be caused by a “deviation” of BK to generate B1R agonists through type I Kininases. Therefore, the combination of increased BK availability and increased expression/activity of type I Kininases and B1R would lead to the pathologic frame illustrated in **Figure 2C**, instead of potentiation of glucose uptake seen in situations of physiological homeostasis, as described in **Figure 2B**.

## Conclusion

According to the data compiled in this review, B2R/eNOS/NO signaling seems to be an amplifying pathway to improve glucose uptake mediated by insulin. Furthermore, this pathway seems to promote substrate oxidation, helping to maintain glycemic control and body weight.

On the other hand, in metabolic diseases, there seems to be some competition with another pathway involving KKS: B1R/iNOS/NO. Therefore, strategies that can prevent the activation of the B1R/NO pathway and preserve B2R/NO would be a good approach to manage metabolic diseases such as diabetes and obesity.

## Data Availability Statement

The original contributions presented in the study are included in the article/**Supplementary Material**; further inquiries can be directed to the corresponding author.

## Author Contributions

MG contributed to bibliographic research, manuscript writing, and hypothesis elaboration. TH contributed to bibliography organization, as well as results and discussion summarization. LM-S contributed with the figures, results summarization, and hypothesis elaboration. MB: results discussion and writing revision. RA: results, discussion, and hypothesis elaboration.

## Funding

This manuscript was suported by the following grants: Fundação de Amparo a Pesquisa do Estado de São Paulo (FAPESP grant 2015/20082-7) and CAPES-PROBRAL (CAPES/DAAD grant 427/15).

## Conflict of Interest

The authors declare that the research was conducted in the absence of any commercial or financial relationships that could be construed as a potential conflict of interest.

## Abbreviations

B2R, kinin B2 receptor; ACEi, Angiotensin-I-Converting Enzyme inhibitor; KKS, kallikrein-kinin-system; B1R, kinin b1 receptor; ACE, angiotensin-I-converting enzyme; SHR, spontaneous hypertensive rat; NO, nitric oxide; STZ, streptozotocin; GLUT 4, glucose transporter type 4; B2KO, kinin b2 receptor knockout mouse; HFD, high fat diet; JNK, c-jun kinase; PKG, cyclic GMP-dependent protein kinase; MKP-5, mitogen-activated protein kinase phosphatase 5; PI3K, phophoinositide 3- kinase; eNOS, endothelial nitric oxide synthase; PKC, protein kinase C; CaMK, calcium-calmodulin-dependent protein kinase; AMPK, AMP-activated protein kinase; cGMP, cyclic guanosine-monophosphate; iNOS, inducible nitric oxide synthase; IRS-beta, insulin-receptor-substrate beta subunit; AKT, protein kinase B; G-alpha-i, GTP-binding protein alpha subunits Gi-Go; NADPH-oxydase, Nicotinamide adenine dinucleotide phosphate oxydase; CPM, carboxypeptidase type M; BK, bradykinin; IRS1, insulin receptor substrate type 1; IR, insulin receptor; KD, kallidin; Plc, phospholipase C; DABK, des-arg-9-bradykinin; DAKD, des-arg-10-kallidin; Src, SRC family tyrosine kinases; Ras, small GTPases; Raf, small GTPases; MEK, mitogen-activated protein-kinase kinase; ERK, extracellular signal-regulated kinase; HOE140, B2 kinin receptor inhibitor; L-NAME, nitric oxide synthase inhibitor.
