# Supplementary material for: Bradykinin B2 Receptor Signaling Increases Glucose Uptake and Oxidation: Evidence and Open Questions
Source: Front Pharmacol. 2020 Aug 4;11:1162. doi: 10.3389/fphar.2020.01162 (PMC7417865; doi:10.3389/fphar.2020.01162)
Supplement: Supplementary file 1 [file DataSheet_1.docx]

**Exclusion Criteria**

**Language:** Original articles published in languages other than English were excluded.

**Non-Original articles:** Reviews, meta-analysis, comments, case reports and editorials fall into this category.

**Out of scope articles:** The purpose of our review is to evaluate the role of B2R signaling in glucose metabolism. Then, we selected articles addressing this topic in the mainly metabolic active tissues: skeletal muscle, white adipose tissue, liver, pancreas, articles evaluating this issue in physical exercise and articles with a “systemic” perspective (Brown adipose tissue is missing because we did not find any article).Therefore, articles related exclusively to other organs, such as heart and kidney, were excluded. Additionally, articles in animal models which did not use any strategy to ensure that the effects were exclusive of B2R (instead of B1R effect) were excluded.

**Number of articles excluded by exclusion criterion:**

**Non-Original articles**: 450

**Language:** 152

**Out of scope:** 1361

**Duplicated articles:** 40

**Total of excluded articles:** 2003
